# Conservation of *Rhodococcus equi* (Magnusson 1923) Goodfellow and Alderson 1977 and rejection of *Rhodococcus hoagii* (Morse 1912) Kämpfer et al. 2014

**DOI:** 10.1099/ijsem.0.004090

**Published:** 2020-04-24

**Authors:** José A. Vázquez-Boland, Mariela Scortti, Wim G. Meijer

**Affiliations:** ^1^​ Microbial Pathogenesis Group, Edinburgh Medical School (Biomedical Sciences - Infection Medicine), University of Edinburgh, Chancellor’s Building, Little France campus, Edinburgh EH16 4SB, UK; ^2^​ UCD School of Biomolecular and Biomedical Science, University College Dublin, Dublin 4, Ireland

**Keywords:** *Rhodococcus equi*, *Rhodococcus hoagii*, *Corynebacterium hoagii*

## Abstract

A recent taxonomic study confirmed the synonymy of *Rhodococcus equi* (Magnusson 1923) Goodfellow and Alderson 1977 and *Corynebacterium hoagii* (Morse 1912) Eberson 1918. As a result, both *R. equi* and *C. hoagii* were reclassified as *Rhodococcus hoagii* comb. nov. in application of the principle of priority of the Prokaryotic Code. Because *R. equi* is a well-known animal and zoonotic human pathogen, and a bacterial name solidly established in the veterinary and medical literature, we and others argued that the nomenclatural change may cause error and confusion and be potentially perilous. We have now additionally found that the nomenclatural type of the basonym *C. hoagii *, ATCC 7005^T^, does not correspond with the original description of the species *C. hoagii* in the early literature. Its inclusion as the *C. hoagii* type on the Approved Lists 1980 results in a change in the characters of the taxon and in *C. hoagii* designating two different bacteria. Moreover, ATCC 7005, the only strain in circulation under the name *C. hoagii *, does not have a well documented history; it is unclear why it was deposited as *C. hoagii* and a possible mix-up with a *Corynebacterium* (* Rhodococcus *) *equi* isolate is a reasonable assumption. We therefore request the rejection of *Rhodococcus hoagii* as a *nomen ambiguum*, *nomen dubium* and *nomen perplexum* in addition to *nomen periculosum*, and conservation of the name *Rhodococcus equi *, according to Rules 56ab of the Code.

A re-examination of the nomenclature of the animal and human pathogen *Rhodococcus equi* in the wake of its recently proposed transfer to a new genus ‘*Prescotella*’ as ‘*Prescotella equi*’ comb. nov. [1, 2] brought back to light the issue of the potential synonymy with *Corynebacterium hoagii* (Morse 1912) Eberson 1918 [3]. We refer for details to the expert analysis by B. Tindall [3], but a brief factual account is as follows. Early evidence that *Corynebacterium equi* (Magnusson 1923) (the basonym of *R. equi* until its transfer to the genus *Rhodococcus* [4]) and *Corynebacterium hoagii* might be heterotypic synonyms came from DNA–DNA hybridization studies reported in 1981 which found that the corresponding types were highly homologous (>88 %) [5]. Results of subsequent numerical taxonomic [6, 7] and mycolic acid composition [8] studies also indicated a close similarity between *R. equi* and the *C. hoagii* type. As a result, *Rhodococcus equi* (Magnusson 1923) Goodfellow and Alderson 1977 and *Corynebacterium hoagii* (Morse 1912) Eberson 1918 were explicitly treated as synonyms in successive editions of *Bergey’s Manual of Systematic Bacteriology* [9–11].

Despite the above, the formal nomenclatural implications of the possibility of *Corynebacterium hoagii* (Morse 1912) Eberson 1918 being an earlier heterotypic synonym of *Rhodococcus equi* (Magnusson 1923) Goodfellow and Alderson 1977 remained unaddressed until very recently. This was in 2014 on the occasion of the description of *Rhodococcus defluvii* sp. nov. by Kämpfer *et al*. [12], which involved polyphasic taxonomic analyses that included the type strains *C. hoagii* DSM 20295^T^ (=ATCC 7005^T^) and *R. equi* DSM 20307^T^ (=ATCC 6939^T^). These studies (i) confirmed the identity of *R. equi* DSM 20307^T^ and *C. hoagii* DSM 20295^T^ as the same species while (ii) they did not find distinct chemotaxonomic differences or 16S rDNA-based phylogenetic separation from other rhodococci to justify the reclassification of *R. equi* into a new genus ‘*Prescotella*’ [12] (consistent with compelling phylogenomic evidence [13, 14]). In application of the principle of priority of the International Code of Nomenclature of Prokaryotes, aka the ‘Prokaryotic code’ [15], Kämpfer *et al*. proposed to reclassify *Corynebacterium hoagii* and *Rhodococcus equi* as *Rhodococcus hoagii* comb. nov., with DSM 20295^T^ (=ATCC 7005^T^=NCTC 10673^T^) as the nomenclatural type [12].

While validly published, the use of the new name *Rhodococcus hoagii* is problematic for a number of reasons. We discussed these in a recent review on *R. equi* [14] and below we summarise the main points and expand on some additional key arguments, for consideration by the Judicial Commission of the International Committee on Systematics of Prokaryotes (ICSP) for rejection of the name *R. hoagii* according to Rule 56a of the Prokaryotic Code [15]. It is beyond this Request for an Opinion to address the issue of the potential illegitimacy of *Rhodococcus* Zopf 1981 (bacterial genus) because a later homonym of the algal genus *Rhodococcus* Hansgirg 1884; for more information about this question we refer to a note by Tindall [16].

First, the *hoagii* epithet has remained largely in disuse, essentially restricted to an obscure type deposited in connection to biotransformative properties (https://www.lgcstandards-atcc.org/products/all/7005, see below), with no obvious link to the identity of *R. equi* as a well-known veterinary pathogen and human opportunistic pathogen [17–19]. The literature that mentions *C. hoagii* prior to its inclusion in the Approved Lists of Bacterial Names 1980 [20] is limited to a few articles on human-associated ‘diphtheroids’ published in the early 1900s [21–23], and to a few taxonomic studies on the ‘coryneforms’ in Japan in the 1970’s where a subculture of the *C. hoagii* type ATCC 7005^T^ was included [24, 25].

In contrast, the *equi* epithet has been in widespread use and constantly associated with its cognate bacterial species since its discovery in 1923 by H. Magnusson as the causative agent of a severe infectious disease of foals [26, 27]. While *R. equi* can also cause opportunistic human infections and colonize other animal species [14, 17, 18, 28–30], it remains best known as a major horse pathogen and thus the epithet is aptly descriptive of the species [31, 32]. Indeed, the name *R. equi* has a solid standing in veterinary and human medicine, animal science and the equine industry, and the change to *R. hoagii* is disconcerting and likely to hamper the traceability and interpretation of the medical, scientific and technical literature regarding this pathogen.

Second, the original descriptions of *C. hoagii* by M.E. Morse in 1912 [22] and F. Eberson in 1918 [2], and by Louis Hoag himself in 1907 [21] for his ‘Organism X’ bacillus (which Morse assumed corresponded to a group of human diphtheroids she commonly isolated, coining for them the epithet ‘*hoagii*’), report features that are difficult to reconcile with the known characteristics of *R. equi* (apart from the faint salmon-pink [21–23] to ‘buff’ pigmentation [21], shared by a number of other bacteria). According to these early descriptions, ‘Organism X’/* C. hoagii* appears to be a relatively common human-associated coryneform/diphtheroid. Hoag found it ‘a number of times’ in throat cultures [21] and Morse refers to these diphtheroids as being the most abundant among the human throat isolates she examined [22]. However, *R. equi* is not known to be a human commensal, only rarely infects people and, when found in human specimens, it is almost invariably associated with severe invasive infections (of suspected exogenous acquisition via exposure to livestock farming environments) [18, 29].

More importantly, furthermore, Hoag [21], Morse [22] and Eberson [23] indicate that ‘Organism X’/* C. hoagii* has the ability to ‘rapidly ferment dextrose (glucose) and saccharose (sucrose)’ with ‘marked acid formation’ as a distinctive characteristic. Yamada and Komagata also report in 1972 for a *C. hoagii* isolate in their collection (AJ 1374, to our knowledge the only other strain apart from ATCC 7005 to have ever been labeled with the name *C. hoagii* after the early descriptions; current availability of this isolate unknown) that it had the ability to produce acid from a variety of sugars (glucose, fructose, mannose, sucrose, maltose, rhamnose, lactose and dextrin) [24]. This is at odds with the known oxidative and eminently asaccharolytic metabolism of *R. equi* [13, 24, 33, 34], the latter linked to the absence of phospho*enol*pyruvate:carbohydrate transport system (PTS) components due to gene loss in the *R. equi */* R. defluvii* monophyletic line of descent [13].

Third, the origin of the *C. hoagii* type kept in several bacterial collections, and its identity with the bacteria described by Hoag and Morse, is uncertain. The strain history at the ATCC repository (https://www.lgcstandards-atcc.org/) indicates that ATCC 7005 was deposited by a F.S. Orcutt at an undisclosed date (affiliation and country of origin also not indicated). It refers to a patent granted in 1958 to Merk Sharp and Dohme on production of diene-steroids by certain corynebacteria, in which ATCC 7005 was applied (Nobile A. Process for production of dienes by corynebacteria. US Patent 2 837 464), but there is no background as to why that particular strain was named *C. hoagii *. The ATCC entry also refers to Nesemann G. *et al*. German Federal Republic Patent 2 302 772 and Canadian Patent 1 022 867 by Hoechst AG from 1973/1974 in relation to a microbiological process of preparation of oxoalkylxanthines; however, this seems to be an error because *C. hoagii* ATCC 7005 is not mentioned at any point in this patent (https://register.dpma.de/DPMAregister/pat/register). The types deposited as DSM 20295^T^ and NCTC 10673^T^ (accession date only specified for the latter, 01/01/1969) are, like the other *C. hoagii* isolates available from other major international repositories (NBRC, JCM, CIP, CCUG), all derivatives of the original F.S. Orcutt’s ATCC 7005, the primary source and history of which, as mentioned, is not documented.

It would therefore appear that the epithet *hoagii* might have been used to designate two different types of bacteria. (i) An undefined sugar-fermenting coryneform/diphtheroid commensal, as originally described by Hoag, Morse and Eberson [21–23] (and Yamada and Komagata for their AJ 1374 isolate [24]); intriguingly, despite being reported by Morse as the ‘largest numerically’ among her collection of human-associated diphtheroids [22], this organism vanishes from the medical literature after the 1920’s, possibly because subsequently recognized under a different name(s) by others. (ii) A *C. equi* isolate probably mistakenly deposited (sometime in the 1950’s) as *C. hoagii* ATCC 7005, which then became the type strain–and only isolate in circulation–of the species [20]. Given the notorious difficulties in differentiating coryneforms in the 1950/60’s and even later, a possible mix-up between two similar-looking cultures is indeed not an implausible scenario. Such mistakes are not uncommon, as illustrated e.g. by the whole genome sequence of the type strain of *Rhodococcus rhodnii* NRRL B-16535^T^ (GenBank accession number GCA_000720375.1), which phylogenetically does not belong to the rhodococci but to an unknown, distant actinobacterium [13, 14].

Taking together all the above, we understand there are sufficient grounds to consider that the name *R. hoagii* under which both *C. hoagii* and *R. equi* have been reclassified meets three of the provisions allowing the Judicial Commission to reject a name according to Rule 56a, namely: (reason 1) an **ambiguous name** (*nomen ambiguum*), i.e. ‘a name which has been used with different meanings and thus has become a source of error’; (reason 2) a **doubtful name** (*nomen dubium*), i.e. ‘a name whose application is uncertain’; and (reason 4) a **perplexing name** (*nomen perplexum*), i.e. ‘a name whose application is known but which causes uncertainty in bacteriology’ [15].

Furthermore, and most importantly, the application of an unfamiliar epithet such as *hoagii* to the well-known animal and human pathogen *R. equi* meets the terms of Rule 56a (reason 5) for rejection: a **perilous name** (*nomen periculosum*), i.e. ‘a name whose application is likely to lead to accidents endangering health or life or both or of serious economic consequences’ [15]. There are indeed real chances of potential misdiagnoses or inaccurate risk assessments, with significant consequences to health and the economy, as a result of the introduction of the new name *R. hoagii* to designate a pathogenic microbe and zoonotic agent with a previously well-established, highly visible and recognizable name.

In an accompanying note to Rule 56a (5) it is stated that ‘if the Judicial Commission recognizes a high order of risk to health, or of serious economic consequences, an Opinion may be issued that the taxon be maintained as a separate nomenspecies, without prejudice to the recognition or acceptance of its genetic relatedness to another taxon’ [15]. It is obvious that this note had in mind the example used to illustrate the application of Rule 56a (5), i.e. *Yersinia pseudotuberculosis* subsp. *pestis *, whereby Opinion 60 [35] places this name in the list of *nomen rejicienda* (rejected) and maintains de facto both *Y. pseudotuberculosis* and *Yersinia pestis* as two separate nomenspecies. The strict application of this interpretation may imply that the names *R. equi* and *R. hoagii* should be maintained as independent nomenspecies. Whereas *R. hoagii* clearly falls under the definition of *nomen periculosum* as given in the Code, the situation is very different from that of *Y. pseudotuberculosis* and *Y. pestis* and we believe there is no basis for the same arrangement.

Thus, while *Y. pestis* evolved as a clone of *Y. pseudotuberculosis* in relatively recent evolutionary times (approximately 1500–10 000 years ago), both form genomically and pathogenically distinct subpopulations [36–39] and warrant being treated as independent nomenspecies. In contrast, *R. hoagii* and *R. equi* are genomically and biologically the same entity in a context in which all isolates of the species are all remarkably genetically homogeneous [13] (Fig. 1). It might be argued that *R. equi* could be applied as a nomenspecies to the equine isolates only, and *R. hoagii* to the rest of the strains, including all isolates from other animal species and humans. However, this possibility cannot be contemplated because the same *R. equi* strain can in principle be found associated with different animal hosts. Indeed, recent evidence indicates that three host-adapted conjugative virulence plasmids, designated pVAPA, pVAPB and pVAPN, each carrying a host-specific variant of the same *vap* pathogenicity island, determine *R. equi* host tropism for, respectively, equines, pigs and ruminants [29, 40, 41]. These virulence plasmids do not appear to be linked to a specific *R. equi* genomic type but to be horizontally exchangeable across the species’ population structure (associated with corresponding host jumps) [13, 29]. In addition, the three plasmid types can also be indistinctly found in isolates recovered from non-adapted (opportunistic) animal hosts (e.g. the case of humans) [14, 28]. Furthermore, the virulence plasmids are often lost in the absence of host selection (e.g. upon *in vitro* culture or during saprophytic life in the environment [28, 29, 42]); this is the case of the *R. equi* type strain ATCC 6939^T^, which lacks the pVAPA plasmid despite originally being an equine clinical isolate [13, 29]). Therefore, maintaining *R. equi* and *R. hoagii* as separate nomenspecies depending on the animal source of (or host-specific plasmid type carried by) the isolate would not only be scientifically unsupported but would potentially lead to further uncertainty and confusion.

**Fig. 1.**
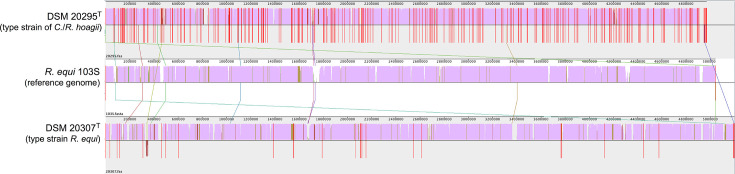
Whole genome sequences of strains DSM 20307^T^ (=ATCC 6939^T^, type strain of *Rhodococcus equ *i; GenBank assembly accession number GCA_002094305.1) and DSM 20295^T^ (=ATCC 7005^T^, type strain of *C*./* R. hoagii *; GenBank assembly accession number GCA_001646645.1) compared with the reference (complete) genome sequence of *R. equi* (*hoagii*) isolate 103S (NCBI RefSeq NC_014659.1) [14]. Alignment reconstructed with Mauve software (http://asap.ahabs.wisc.edu/mauve/). Similarity plot is in pink, height indicates level of similarity; plot pointing downwards indicates similarity to the reverse strand of the genome. Blank regions represent fragments that were not aligned or contain genome-specific sequences. Red vertical divisions indicate contigs. Note the dominant solid pink sequence segments which denote near complete conservation between the genomes, consistent with the high degree of genomic relatedness between *R. equi* (*hoagii*) isolates [13]. Pairwise Average Nucleotide Identity (ANI): 103S/DSM 20295=98.85 %, 103S/DSM 20307=99.14 % (calculated with FastANI 1.3).

Some of the points put forward here regarding the application of an unfamiliar epithet to a well-known pathogen have been previously raised by G. Garrity in a Request for an Opinion to the Judicial Commission for the conservation of *R. equi* and rejection of *Corynebacterium hoagii* as a perilous name [43]. However, since then *Corynebacterium hoagii* and *R. equi* have been officially reclassified as *Rhodococcus hoagii* comb. nov. by Kämpfer *et al*. [12], and this nomenclatural act included in a Notification List [44]. We have now also identified significant additional issues regarding the *R. hoagii* type ATCC 7005^T^ which cast reasonable doubts as to whether it indeed corresponds to the same organism that Morse and Eberson described as *C. hoagii* in 1912/1918 [22, 23], formally setting the priority of the name. To recapitulate:

1. The nomenclatural type bearing the epithet *hoagii*, ATCC 7005 (the only strain in circulation at the time that was named *C. hoagii *), does not correspond to the properties of *C. hoagii* originally reported by Morse (1912) and Eberson (1918) – and reproduced in Bergey’s Manual editions until 1974.
2. The designation of ATCC 7005 as the *C. hoagii* type in the Approved Lists 1980 results in a change in the characters of the taxon.
3. ATCC 7005 does not have a well documented history, its origin is uncertain, and it is unclear why it was named *C. hoagii *. A likely scenario is that the name *C. hoagii* was arbitrarily or accidentally coopted in the 1950’s to deposit under ATCC 7005 a bacterium which actually was a *C. equi* isolate (perhaps as a result of a culture mix-up, in those times *C. equi* was already commonly represented in corynebacterial collections).
4. The name *C. hoagii* has clearly been used to designate two different bacteria, an undefined human-associated glucose- and sucrose-fermenting diphtheroid (of which to our knowledge there is currently no reference culture available), and the animal and zoonotic pathogen *R. equi *.

Consequently, we are hereby submitting a Request for an Opinion to the Judicial Commission for the conservation of *Rhodococcus equi* (Magnusson 1923) Goodfellow and Alderson 1977 as the correct name of the species, and rejection of *Rhodococcus hoagii* (Morse 1912) Kämpfer et al. 2014 as a *nomen ambiguum*, *nomen dubium* and *nomen perplexum* in addition to *nomen periculosum*, according to Rules 56ab of the Prokaryotic Code [15].

If the Judicial Commission takes into consideration this request and declares *R. hoagii* as a rejected name, a simple and practical solution to resolve the nomenclatural conundrum around *R. equi* would be to recognize the lack of proper documented evidence and potential mistake and permanent source of error with the ATCC 7005 deposition, and reclassify the nomenclatural type originally bearing the epithet *hoagii* as *R. equi* ATCC 7005. Since the type of the basonym, *C. equi* ATCC 6939^T^, was deposited earlier, the *R. equi* type should revert to this strain. In contrast to ATCC 7005, ATCC 6939 has a well-documented history; it derives from NCTC 1621, an original equine clinical isolate from H. Magnusson who discovered the species in 1923.

**Note added in proof**

The Bergey’s Manual also indicates in its 7th (1957) and 8th (1974) editions that *Corynebacterium hoagii* produces on agar culture “small”, “granular”, “entire” colonies, inconsistent with the characteristic heavy growth with moist, smooth, glistening colonies of *R. equi *. This further highlights the change in the characters of the taxon that results from designating ATCC 7005 (a *bona fide R. equi* isolate) as the type strain of *C. hoagii* in the Approved Lists 1980.
